# Serial Changes in Body Composition and the Association with Disease Activity during Treatment in Patients with Crohn’s Disease

**DOI:** 10.3390/diagnostics12112804

**Published:** 2022-11-15

**Authors:** Ji Young Lee, Kyung Won Kim, Yousun Ko, Chi Hyuk Oh, Bo Hyun Kim, Seong Jin Park, Myung-Won You

**Affiliations:** 1 Department of Radiology, Kyung Hee University College of Medicine, Kyung Hee University Hospital, Seoul 02447, Republic of Korea; 2 Department of Radiology and Research Institute of Radiology, University of Ulsan College of Medicine, Asan Medical Center, Seoul 05505, Republic of Korea; 3 Biomedical Research Center, Asan Institute for Life Sciences, Asan Medical Center, Seoul 05505, Republic of Korea; 4 Department of Gastroenterology, Kyung Hee University College of Medicine, Kyung Hee University Hospital, Seoul 02447, Republic of Korea; 5 Department of Radiology, Seoul St. Mary’s Hospital, College of Medicine, The Catholic University of Korea, Seoul 06591, Republic of Korea

**Keywords:** body composition, Crohn’s disease, disease activity, remission

## Abstract

Objectives: To analyze serial changes in body composition and investigate the association between body composition changes and disease activity changes in patients with Crohn’s disease (CD). Methods: Seventy-one patients with CD who had been treated and followed-up at our institution were included. Two to four computed tomography images were acquired at baseline, and the 2–5-year, 5–8-year, and last follow-ups were selected per patient for body composition and disease activity analyses. Visceral fat area (VFA), skeletal muscle index (SMI; skeletal muscle area/height^2^), and subcutaneous fat area (SFA) were assessed using an artificial-intelligence-driven fully automated method. Disease activity was assessed using a modified computed tomography scoring system and the Simple Endoscopic Score for Crohn’s Disease. The associations between body composition, disease activity, and remission were investigated. Results: The mean age was 29.83 ± 11.27 years; most patients were men (48/71, 67.6%); and the median follow-up was 144 (12–264) months. Overall, VFA and SFA gradually increased, while SMI decreased during the follow-up. Sarcopenia was associated with the female sex, higher disease activities at baseline (*p* = 0.01) and the last follow-up (*p* = 0.001). SMI and SFA inversely correlated with the disease activity, i.e., the more severe the disease activity, the lower the SMI and SFA (*p* < 0.05). SMI at the last follow-up was the only significant predictor of remission (OR = 1.21, 95% confidence interval: 1.03–1.42, *p* = 0.021). Conclusion: SMI decreased while VFA and SFA increased during the treatment follow-up in patients with CD. Sarcopenia was associated with higher disease activity, and SMI and SFA inversely correlated with disease activity. SMI at the last follow-up was the significant factor for remission.

## 1. Introduction

Body composition analysis, which typically refers to the quantification of body fat and muscle mass, is of great interest to medical professionals [1]. In vivo measurements using diagnostic imaging are now considered the gold standard for body composition assessment. Computed tomography (CT) is a useful tool for evaluating muscle and fat in the body, providing accurate measurements of the area and volume of muscle and fat based on CT attenuation values.

Imaging-based body composition analysis has been studied with respect to a range of medical conditions (e.g., obesity and its associated problems) [2], various cancers [3,4], and morbidity or mortality rate after major surgeries [5,6]. Several studies have reported that excessive body fat is related to increased morbidity and mortality in patients with different pathologies [2]. Recently, sarcopenia (i.e., loss of skeletal muscle mass) was found to be a negative predictor in both healthy and diseased states [7,8].

Crohn’s disease (CD) is an inflammatory bowel disease that progresses as a result of recurrent attacks [9]. Several previous studies have described changes in the body composition of patients with CD. Malnutrition, a common condition encountered in patients with CD, is often associated with sarcopenia. Recent data suggest that the prevalence of sarcopenia in patients with CD is approximately 50–70%; however, a degree of heterogeneity exists in CD cohorts [10,11]. Visceral adiposity is an important factor in the pathogenesis of CD, both as a cause and consequence of the evolution of the disease, and has been reported to be increased patients with CD [12,13]. A number of studies have found that a high visceral fat area (VFA) is correlated with higher morbidity or postoperative complications [14,15], although there have been controversial results showing no correlation between VFA and disease behavior [16] or adverse outcomes in patients with CD [17].

In this study, we aimed to analyze the serial changes in body composition in patients with CD during a long period of treatment and to evaluate the association between body composition and disease activity.

## 2. Materials and Methods

### 2.1. Patients

This retrospective study was approved by the institutional review board of our institution (approval no. 2020-09-029-003), which waived the requirement for informed consent. We searched the electronic medical records and images (from the picture archives and communication system) of patients diagnosed with CD between January 2000 and December 2009 who were followed up until August 2020. The diagnosis of CD was determined according to a clinical evaluation and a combination of biochemical, radiologic, endoscopic, and histologic investigations [18,19]. Patients who (a) were initially diagnosed with CD at our institution, (b) received treatments and were regularly monitored for treatment response, (c) underwent at least two or more CT examinations (at baseline and at one or more follow-up time points after treatment), and (d) had identifiable treatment regimens were included in this study. Patients who lacked information on treatment regimen, were lost to follow-up after the initial visit, and did not undergo follow-up CT after baseline examination were excluded (Supplementary Figure S1).

### 2.2. Clinical, Radiologic, and Endoscopic Data Analyses

We recorded the age at diagnosis, sex, body mass index (BMI), follow-up period, and treatment regimen of each patient. BMI was calculated as weight divided by height squared. Two reviewers (with 15 and 5 years of experience in abdominal radiology, respectively) assessed baseline and follow-up CT examinations by consensus. Follow-up CT examinations were selected from three time intervals: 2–5 years, 5–8 years, and after 8 years. The reviewers assessed the disease activity in each CT examination using a modified CT scoring system, which we developed for our own study (Table 1). Five CT parameters were assessed: (a) bowel wall thickness, (b) intensity of bowel wall enhancement, (c) pattern of bowel wall enhancement, (d) lesion length, and (e) comb sign. The four CT parameters were scored from 0 to 3. (a) Maximum bowel wall thickness was measured and scored as follows: ≤3 mm (score 0), 4–5 mm (score 1), 6–7 mm (score 2), and ≥8 mm (score 3). (b) Bowel wall enhancement was evaluated compared to adjacent vessels and scored as follows: normal (score 0), mild (score 1), moderate (score 2), and severe (score 3). (c) The pattern of bowel wall enhancement was classified as homogeneous if one-layered enhancement was observed (score 1). Bilaminar and trilaminar enhancement were classified as mucosal (score 2) and layered (score 3) enhancement, respectively. (d) The lesion length was scored according to the sum of the lengths of active lesions, as follows: none (score 0), <5 cm (score 1), 5–15 cm (score 2), and ≥15 cm (score 3). (e) The comb sign was defined as engorged vasa recta (score 1). If there were multiple lesions, the most severe lesion was selected. Disease activity was classified as none (no signs of active disease), mild (score 6≥, no features with score 3), moderate (scores 7–10 or presence of any one feature with score ≥ 3), and severe (total score ≥ 11), according to the total CT score. Cases with at least one complication (abscess, fistula, or stenosis) were classified into the severe activity category.

One gastroenterologist (with 15 years of experience in colonoscopy) reviewed colonoscopic images using the Simple Endoscopic Score for Crohn’s Disease (SES-CD) to retrospectively assess endoscopic disease activity. Disease activity was classified as follows: normal (remission), SES-CD < 4; mild, SES-CD 4–6; moderate, SES-CD 7–15; and severe, SES-CD > 16 [20].

### 2.3. CT Techniques

CT examinations were performed using one of the following multidetector CT scanners: 16-channel Lightspeed (GE Healthcare, Milwaukee, WI, USA; n = 77), 16-channel Somatom Sensation (Siemens Healthineers, Erlangen, Germany; n = 17), 64-channel Brilliance (Philips, Amsterdam, the Netherlands; n = 41), 64-channel Aquilion (Toshiba Medical Systems, Otawara, Japan; n = 44), and 128-channel Ingenuity (Philips, n = 54). Among 232 CT examinations, 157 were performed according to the small-bowel enterography protocol, as follows: 1.5 L of 4% sorbitol was ingested by the patient 30–45 min before the examination, and abdominopelvic CT images were acquired during the portal venous (70 s after injection) and delayed (3 min after injection) phases following the injection of a nonionic contrast medium (iomeprol 300; Bracco Co., Ltd., Milan, Italy). A total of 1.8–2.0 mL/kg body weight was injected at a rate of 2.4 mL/s with a 20 mL flush of normal saline after contrast injection. The scanning parameters were as follows: peak voltage, 120 kVp; tube current–time product, 150–200 mAs with automated tube current modulation; slice thickness, 2.5–5 mm with a 2.5–5 mm reconstruction interval; field of view, 300–380 mm; gantry rotation time, 0.5–0.6 s; detector configuration, 0.625 mm; z-axis coverage, 24, 40, and 40 mm; pitch, 0.9, 0.7, and 0.8 s; table speed, 43.2, 47.5, and 63.8 mm/s; and single breath-hold helical acquisition time, 9–10 s for 16-, 64-, and 128-channel CT examinations.

### 2.4. Body Composition Analyses

The same CT images used in the CT activity assessment were selected for body composition analyses. The inferior endplate level of the L3 vertebra was chosen as the measurement level. From this single-slice image, specific tissue demarcation was performed based on Hounsfield units (HU) using an artificial-intelligence-driven fully automated segmentation technique (AID-U™; iAID Inc., Seoul, Republic of Korea). The skeletal muscle area (SMA, cm^2^), including all muscles on the selected axial images (i.e., psoas, paraspinal, transversus abdominis, rectus abdominis, quadratus lumborum, and internal and external obliques), was demarcated using predetermined thresholds (−29 to +150 HU). The VFA (cm^2^) and subcutaneous fat area (SFA, cm^2^) were also demarcated using fat tissue thresholds of −190 to −30 HU. The SMA was adjusted by the square of the height (SMA/height^2^) to obtain the skeletal muscle index (SMI, cm^2^/m^2^). Sarcopenia was defined as SMI < 38.5 cm^2^/m^2^ in women and <52.4 cm^2^/m^2^ in men [21].

### 2.5. Outcome Analyses

Disease activity was defined according to CT activity scores and SES-CD. Remission was defined as SES-CD <4 and the absence of clinical symptoms. We compared changes in body composition according to disease activity and the presence of remission during the treatment follow-up.

### 2.6. Statistical Analyses

Continuous variables are presented as mean values with standard deviations, and categorical variables are presented as percentages. Categorical and continuous data were compared using the chi-square or Fischer’s exact test and Student’s *t*-test, respectively. Serial changes in body composition and the association between CT activity and body composition or remission were analyzed using a linear mixed model. A logistic regression analysis was performed to evaluate significant variables predicting remission. All statistical analyses were performed using SAS (version 9.4; SAS Institute, Cary, NC, USA) and R package version 4.2.0. Statistical significance was set at *p* < 0.05.

## 3. Results

### 3.1. Clinical Characteristics of the Study Population

Of the 84 identified patients, 13 were excluded and a total of 71 patients with CD were included in this study. The demographic and clinical characteristics of the enrolled patients are listed in Supplementary Table S1. The mean age of the patients was 29.83 ± 11.27 years; 48 patients were men (67.61%); the mean BMI was 19.78 ± 2.94 kg/m^2^; and the median follow-up period was 144 months (range 12–264 months). At baseline, most of the patients had active inflammation on endoscopy (95.65%), and this proportion decreased during follow-up (62.96%, 56.82%, 37.21%). Similarly, most of the patients had moderate to severe CT scores at baseline (88.7%), and this proportion gradually decreased. Most of the patients received biologic treatment (81.6%), and a minority received immunosuppressant-only treatment (14%) or aminosalicylate-only treatment (4.2%). Twenty-four patients showed remission at the last follow-up (33.8%).

### 3.2. Serial Changes in Body Compositions and Disease Activity

**Figure 1:**
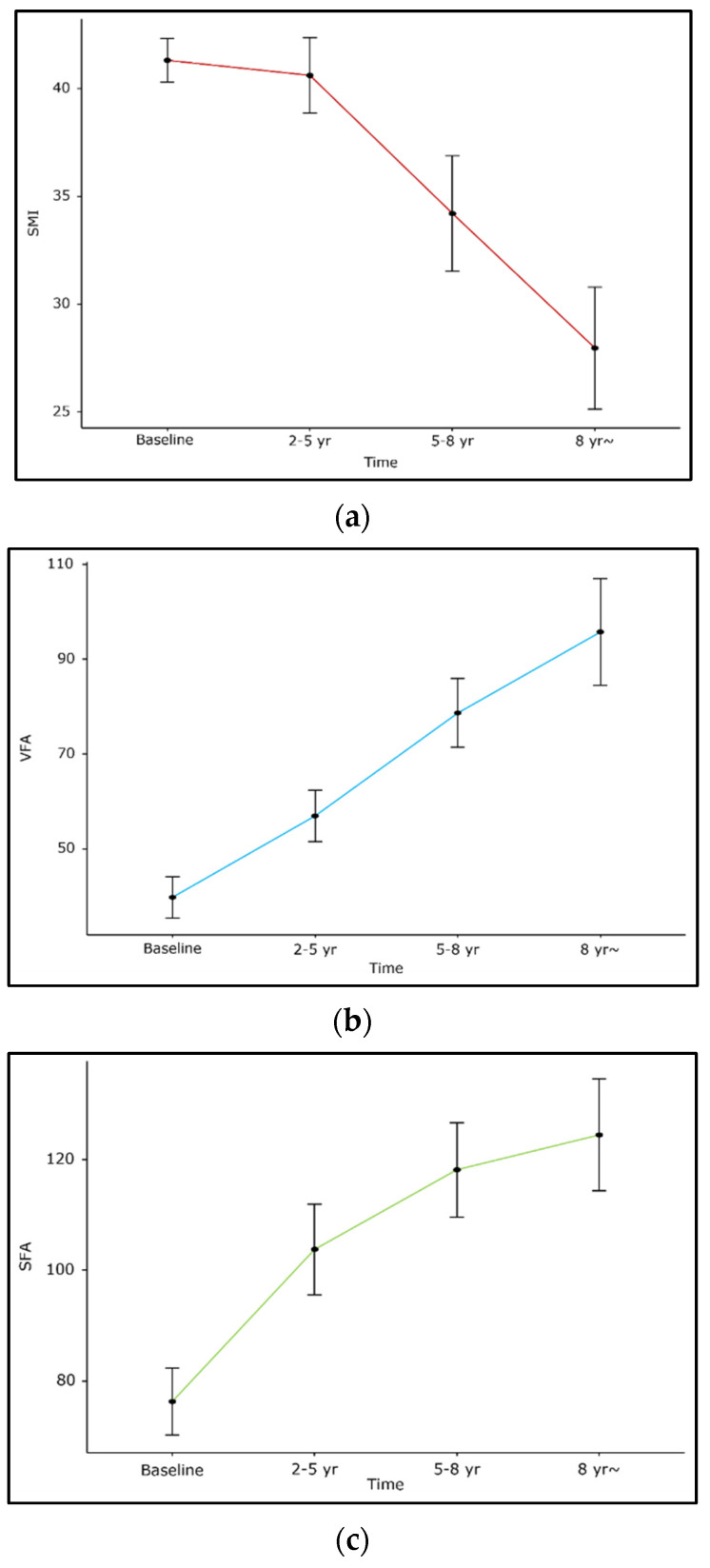
Changes in body composition during treatment follow-up in patients with Crohn’s disease. (**a**) SMI, (**b**) VFA, (**c**) SFA.

Overall, SMI decreased during the treatment follow-up period (beta −1.8, 95% confidence interval [CI]: −2.6~−1.0, *p* < 0.001), while SFA and VFA increased (beta 6.5, 95% CI: 4.6~8.5, *p* < 0.001; 7.2, 95% CI: 4.9~9.4, *p* < 0.001, Figure 1). The BMI of the included patients showed a slight decrease (beta −0.8, 95% CI: −1.2~−0.4, *p* < 0.001).

Both CT activity scores and SES-CD gradually decreased during the follow-up period, showing a decreased disease activity; the proportions of normal and mild activity grades increased, whereas those of moderate and severe disease activity grades decreased (Supplementary Figure S2).

### 3.3. Association of Body Composition with Disease Activity

Thirty-eight (53.5%) patients were classified into the sarcopenia group according to predefined criteria. Women represented sarcopenia more frequently than men (55.3% vs. 6.5%, *p* < 0.001). Baseline CT activity grades were higher in the sarcopenia group than in the non-sarcopenia group (larger proportions of moderate and severe grades; 97.4% vs. 80.7%, *p* = 0.01). SES-CD at the last follow-up was higher in the sarcopenia group than in the non-sarcopenia group (larger proportions of mild and moderate grades with less normal grades; 69.6% vs. 16.7%, *p* = 0.001, Table 2).

**Figure 2:**
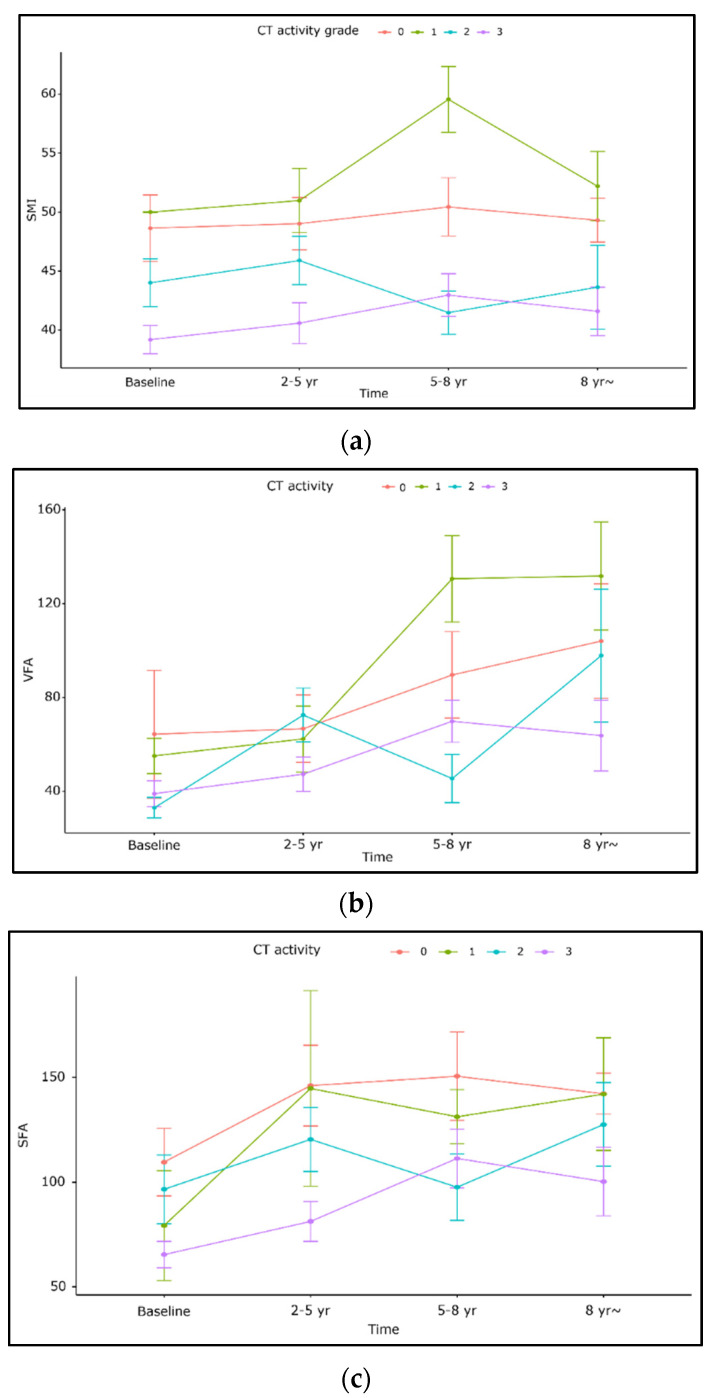
Changes in body composition stratified by CT activity grades. (**a**) SMI, (**b**) VFA, (**c**) SFA.

According to the analysis of body composition stratified by disease activity, SMI decreased as disease activity increased per both CT activity scores and SES-CD. SMI was significantly lower in moderate (beta-4.1 95% CI: −7.9~−0.35, *p* = 0.032) and severe (−6.3, −10~−2.7, *p* < 0.001) CT grades compared to normal grades. SMI was also lower per mild and moderate (−4.2, −7.2~−1.2, *p* = 0.007) and severe (−5.5, −8.7~−2.2, *p* = 0.001) SES-CD than in normal endoscopic activity. SFA showed a similar pattern to those of SMI; SFA decreased as increased disease activity per both CT scores and SES-CD (*p* < 0.05). VFA showed no significant association with disease activity scores (Table 3, Figure 2). VFA/SFA ratio was additionally analyzed and showed no significant association with disease activity (Supplementary Table S2).

### 3.4. Association of Body Composition with Remission

**Figure 3:**
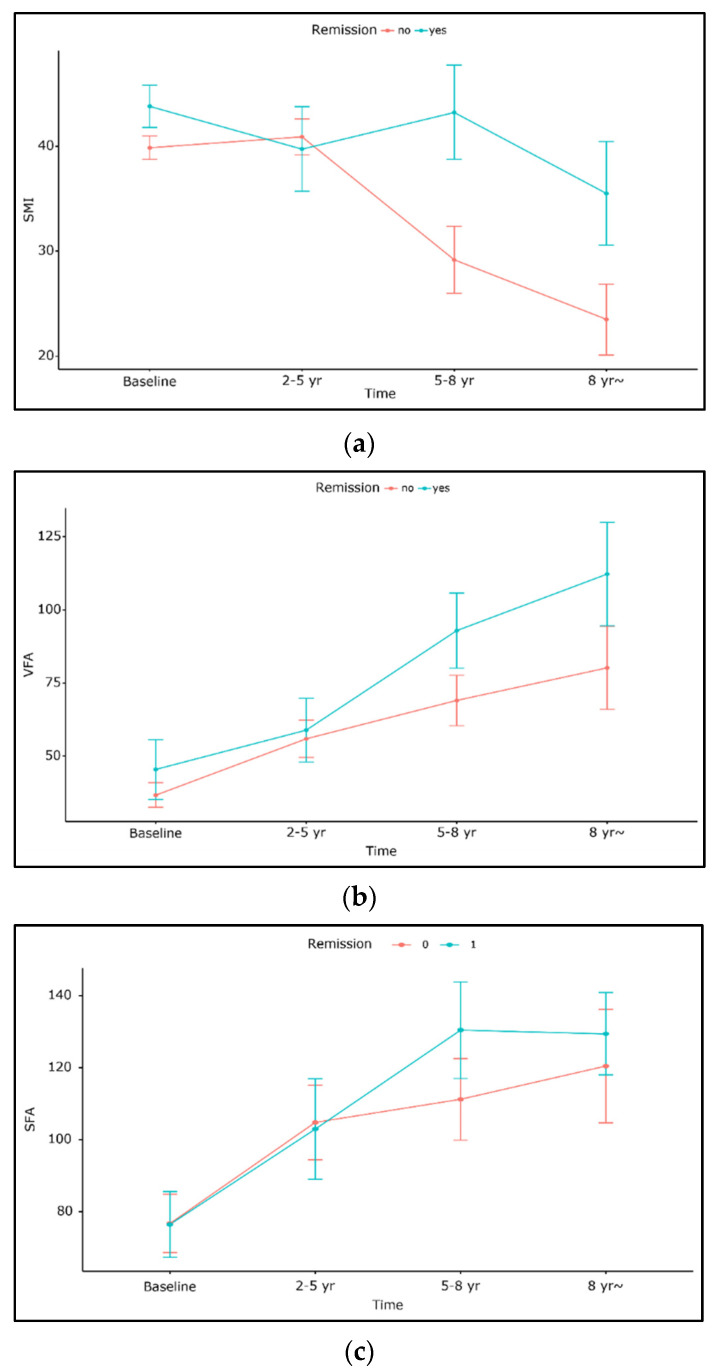
Comparison of changes in body composition according to presence or absence of remission. (**a**) SMI, (**b**) VFA, (**c**) SFA.

None of the three parameters, i.e., SFA, VFA, or SMI, significantly differed with the presence of remission. VFA and SFA increased irrespective of remission, with a slightly greater increase in the remission group, although without statistical significance. SMI was slightly higher in the remission group (*p* = 0.61, Figure 3). In the logistic regression analysis, SMI at the last follow-up was the only predictor of remission (OR = 1.21, 1.03–1.42, *p* = 0.021). Baseline sarcopenia, VFA, and SFA at last follow-up were not significant variables for predicting remission (Table 4).

## 4. Discussion

Previous studies reported that patients with CD have lower muscle and fat mass than healthy individuals [22]. This may be explained by reduced dietary intake, malabsorption, and metabolic disturbances that cause losses of glucose, protein, and fat [23,24]. Altered body composition in CD (increased VFA or decreased muscle mass) is associated with complicated CD [21,25], adverse postsurgical outcomes [26,27], and poor response to biologics [28]. In this study, sarcopenia and lower SMI and SFA were associated with higher disease activity but not with complications, surgery rate, or regimen change.

In this study, disease activity assessed in both CT and colonoscopy showed inverse correlations with SMI and SFA. SMI and SFA were lower with moderate and severe disease activities than with normal and mild disease activities, consistent with a previous study [29] reporting that disease severity could affect the degree of muscle depletion, i.e., more severe disease activity corresponds to greater muscle depletion.

Rocha et al. studied changes in fat and muscle mass during active and remission phases [29]. They reported that both fat and muscle depletion occurred in the active phase, and although partial recovery of fat mass occurred during remission, muscle mass depletion remained, consistent with our study showing increased body fat (VFA, SFA) and decreased muscle (SMI) during follow-up after treatment. Moreover, less muscle depletion and SMI at the last follow-up were predictors of remission in our logistic regression analysis. In the active phase of CD, a decrease in oral intake and mucosal inflammation with associated diarrhea occur, leading to a loss of nutrients. Furthermore, proinflammatory cytokines have a direct catabolic effect on protein metabolism, reducing anabolic drive [11,29]. These factors may be attributed to muscle and fat mass depletions. The increase in body fat during remission may be associated with an improved nutritional status resulting from improved bowel condition as well as decreased bowel inflammation and its catabolic effects.

Visceral adipose tissue (VAT), especially mesenteric adipose tissue (MAT), has a significant impact on the clinical course and therapeutic outcome of CD. MAT is linked to pro-inflammatory immune mediators that interact with the impaired intestinal epithelial barrier and translocation of gut microflora, leading to bowel inflammation [30]. In contrast, MATs have potential anti-inflammatory and protective roles as a fourth barrier in the local host defense system [31]. Previous studies indicated that the mesenteric fat area (MFA), MAT/VAT volumes, mesenteric fat index (MFI), or ratio of VFA to SFA correlate with disease activity measured by the CD activity index (CDAI) and C-reactive protein (CRP), endoscopic scores, complications, or disease recurrence [13,32,33,34]. However, in our study, VFA and the VFA/SFA ratio showed no significant correlation with disease activity, whereas SFA showed an inverse correlation. This finding highlights the different characteristics of VFA and SFA in terms of metabolic and immunological profiles [35]. VFA increased as disease activity decreased during treatment follow-up, which might be partly attributed to mesenteric fatty proliferation near the diseased bowel segment, represented by creeping fat. Creeping fat is defined as an increase in fat appearing as a blank space that results in the separation or displacement of the adjacent bowel loops [36]. This accumulation in mesenteric fat correlates with the degree of bowel inflammation and fibrosis [37]. However, it has a more complex role as a regulator of bowel disease, considering that VFA, including creeping fat, increases regardless of disease activity in our study population.

Sarcopenia frequently arises from chronic inflammation of CD and is associated with a risk of disability [38,39]. In this study, sarcopenia was associated with increased disease activity but not with adverse outcomes, such as complications, poor treatment response, or the surgery rate. Although baseline sarcopenia was not a significant factor for remission, less muscle depletion at the last follow-up was significantly associated with remission. Further studies with larger populations are required to obtain more consolidated evidence of prognostic significance of body fat and muscle changes in patients with CD.

This study has several limitations. First, this study has the inherent limitation of being a retrospective study. As the initial date of diagnosis was far from the present date, the collected medical information was incomplete. However, we focused on analyses of body composition and disease activity on selected CT and colonoscopy images, which provided objective data represented by numerical values. Second, the CT protocols and vendors were heterogeneous and inconsistent among the included patients. However, CT images deemed inadequate for body composition analyses were excluded during the analysis using the fully automated segmentation technique. Third, we used a modified CT scoring system that has not been fully validated. As no credibly validated CT scoring system is available, we developed a scoring system that can be applied to CT images with reference to previous CT scoring systems [40] and the commonly used magnetic resonance scoring system [41]. Moreover, we added SES-CD to complement the modified CT scoring system. Crohn’s disease activity index (CDAI), which is a well-recognized parameter of measuring disease activity and widely used in clinical trials and clinical practice, was not used in this study [42]. However, CDAI has several limitations, including interobserver variability, subjective interpretation (“general well-being” and “abdominal pain intensity”), inconvenient reporting of stool frequency (based on a diary filled in by the patient for seven days), limited application in patients with fistulizing and stenosing disease, and patients with previous extensive ileocolic resection or stoma [43]. We believe combined radiologic and endoscopic assessments can fulfill proper disease activity assessment. Fourth, the included patients had heterogeneous treatment regimens. Most patients received biologics, and a small proportion received immunosuppressant- or aminosalicylate-only treatment without biologics (18.3%, 13/71). We included these patients because our study aimed to evaluate the overall trend of changes in body composition during treatment regardless of the specific response to treatment regimens.

## 5. Conclusions

Patients with CD showed a gradual increase in fat and a decrease in muscle mass as the disease activity decreased during the follow-up after treatment. Sarcopenia was associated with higher disease activity, and SMI inversely correlated with disease activity. SMI at the last follow-up was a significant factor for remission.

## Figures and Tables

**Table 1:** Modified CT scoring system.

	0	1	2	3
Wall thickness	3 mm≥	4–5 mm	6–7 mm	≥8 mm
Wall enhancement (compared to adjacent vessels)	Normal	Mild; minor increase, but less than vessels	Moderate; moderate increase but less than vessels	Severe; marked increase, similar to vessels
Enhancement pattern	Normal	^§^ Homogenous	^¥^ Mucosal	^£^ Layered
Lesion length	None	5 cm	5–15 cm	15 cm≤
Comb sign	No	Yes		
None: no signs of active disease Mild: total scores ≤6 or no features with score 3 Moderate: total scores 7–10 or presence of any one feature ≥3 Severe: total scores ≥11 or presence of at least one complication including abscess, fistula or stenosis				

^§^ one-layered bowel wall with enhancement. ^¥^ two-layered bowel wall with inner layer enhancement. ^£^ three-layered bowel wall with inner and outer layer enhancements and hypo-enhancement of the middle layer.

**Table 2:** Comparison of variables according to baseline sarcopenia.

Variables	No Sarcopenia (n = 31)	Sarcopenia (n = 38)	*p*-Value
Sex (%) M F	29 (93.5) 2 (6.5)	17 (44.7) 21 (55.3)	<0.001
Age	30.2 ± 9.8	29.3 ± 12.7	0.738
Smoking (%)	15 (48.3)	8 (21)	0.065
Follow-up period (months)	138.7 ± 60.2	131.5 ± 59.3	0.617
Baseline CT activity (%) Normal Mild Moderate Severe	4 (12.9) 2 (6.5) 10 (32.3) 15 (48.4)	1 (2.6) 0 (0.0) 6 (15.8) 31 (81.6)	0.01
Baseline SES-CD	11.8 ± 7.3	13.9 ± 7.7	0.275
2–5 y SES-CD	8.8 ± 6.8	9.8 ± 7.1	0.586
5–8 y SES-CD	3.5 ± 3.6	6.3 ± 6.2	0.087
8 y last SES-CD	4.6 ± 6.2	7.3 ± 5.8	0.158
8 y last SES-CD (%) Normal Mild/Moderate Severe	14 (77.8) 3 (16.7) 1 (5.6)	6 (26.1) 16 (69.6) 1 (4.3)	0.001
Systemic steroid IV (%) No Yes Unknown	27 (93.1) 1 (3.4) 1 (3.4)	34 (89.5) 2 (5.3) 2 (5.3)	1
Change in regimen from IMS to biologics (%)	24 (88.9)	28 (80.0)	0.491
Change in the biologics regimen (%)	10 (40.0)	13 (39.4)	1
Dose optimization (%)	12 (46.2)	13 (39.4)	0.798
Remission (%)	13 (43.3)	10 (26.3)	0.224
Surgery (CD-associated) (%)	5 (16.1)	9 (23.7)	0.635
Time to surgery (months	51.9 ± 43.5	58.8 ± 79.3	0.813
Complications (%)	9 (29.0)	12 (31.6)	1

**Table 3:** Association of body composition with CT activity scores or SES-CD scores.

(1) SMI		
Characteristics	Beta (95% CI)	*p*-Value
Time	0.33(−0.41~1.1)	0.38
Sex F M	- 10(6.6~13)	<0.001
CT activity Normal Mild Moderate Severe	- 0.7(−4.6~6.0) −4.1(−7.9~−0.35) −6.3 (−10.0~−2.7)	0.79 0.032 <0.001
Time × CT activity 2–5 y f/u 5–8 y f/u >8 y f/u	0.16(−0.91~1.2) 0.13(−0.77~1.0) 0.52(−0.30~1.3)	0.77 0.77 0.21
**(2) VFA**		
Time	0.33 (−0.41~1.1)	0.38
Sex F M	- 1.9 (−16~20)	0.84
SES-CD Normal Mild/moderate Severe	- −0.43 (−13~12)−11 (−27~5.5)	0.95 0.20
CT activity Normal Mild Moderate Severe	- −0.30 (−35~35) −19 (−44~6.1) −13 (−37~11)	0.99 0.14 0.28
Time × CT activity 2–5 y f/u 5–8 y f/u >8 y f/u	0.41 (−7.1~7.9) 2.2 (−4.4~8.9) −1.0 (−7.0~5.0)	0.91 0.51 0.75
**(3) SFA**		
Time	0.70 (−4.9~6.3)	0.81
Sex F M	- −31 (−56~−6.5)	0.014
CT activity Normal Mild Moderate Severe	- −16 (−59~26) −44 (−75~−14) −48 (−76~−20)	0.45 0.004 0.001
Time × CT activity 2–5 y f/u 5–8 y f/u >8 y f/u	3.1 (−5.4~12) 6.7 (−0.43~14) 5.9 (−0.39~12)	0.48 0.065 0.066

**Table 4:** Logistic regression analysis predicting remission.

	Univariate OR (95% CI)	*p*-Value	Multivariate OR (95% CI)	*p*-Value
Sex: F vs. M	0.77 (0.27,2.25)	0.635	19.57 (0.62,617.03)	0.091
Age	0.99 (0.94,1.03)	0.529	0.96 (0.86,1.06)	0.388
Baseline sarcopenia	0.47 (0.17,1.3)	0.144	0.19 (0.01,3.34)	0.258
Baseline BMI	1.07 (0.9,1.27)	0.438	0.65 (0.36,1.16)	0.145
SMI at the last follow-up	1.02 (1,1.05)	0.048	1.21 (1.03,1.42)	0.021 **
VFA at the last follow-up	1.01 (1,1.02)	0.164	1 (0.98,1.02)	0.811
SFA at the last follow-up	1 (0.99,1.01)	0.669	1 (0.98,1.02)	0.959

** means *p* < 0.05.

## Data Availability

The data presented in this study are available on request from the corresponding author. The data are not publicly available due to privacy.

## Supplementary Materials

The following supporting information can be downloaded at: https://www.mdpi.com/article/10.3390/diagnostics12112804/s1, Figure S1: flow chart of patient selection, Figure S2: changes in disease activity in CT activity grades and SES-CD scores, Table S1: Clinical characteristics of study population (n = 71), Table S2: Association of VFA/SFA ratio with CT activity scores and SES-CD scores.
