# Supplementary material for: Serial Changes in Body Composition and the Association with Disease Activity during Treatment in Patients with Crohn’s Disease
Source: Diagnostics (Basel). 2022 Nov 15;12(11):2804. doi: 10.3390/diagnostics12112804 (PMC9689369; doi:10.3390/diagnostics12112804)
Supplement: Supplementary file 1 [file diagnostics-12-02804-s001.zip › diagnostics-1984122-supplementary.pdf]

Supplementary Table S1. Clinical characteristics of study population (n=71).

**Variables**	**N(%)**
Sex (%)<br>F<br>M	 23 (32.4)<br>48 (67.6)
Age	29.8 ± 11.3
Follow-up period (mo, median [IQR])	144.0 [97.0, 180.5]
Prior surgery (%)	6 (8.6)
Prior perianal surgery (%)	17 (24.3)
Smoking (%)	25 (35.8)
Baseline sarcopenia (%)	38 (55.1)
Baseline CT activity (%)<br>Normal<br>Mild<br>Moderate<br>Severe	 5 (7.0)<br>2 (2.8)<br>16 (22.5)<br>48 (67.6)
Baseline SES-CD score (%)<br>Normal<br>Mild/Moderate<br>Severe	 11 (16.2)<br>32 (47.1)<br>25 (36.8)
Change of IMS to biologics (%)	53 (82.8)
Change of biologics (%)	24 (40.7)
Dose optimization (%)	26 (43.3)
Remission	24 (34.3)
Surgery	15 (21.1)
Time to surgery (mo)	64.5 ± 68.6
Complication (%)<br>None<br>Stenosing<br>Penetrating<br>Combined stenosing and penetrating	 50 (70.4)<br>16 (22.5)<br>1 (1.4)<br>4 (5.6)

Supplementary Table S2. Association between VFA/SFA ratio with CT activity scores and SES-CD scores.

**Characteristics**	**Beta (95% CI)**	**p-value**
time	0.03 (0.01~0.06)	0.004
Sex		
F	-	
M	0.28(0.09~0.47)	0.005
SES-CD		
Normal	-	
Mild/moderate	0.03 (-0.1~ 0.16)	0.68
Severe	0.01 (-0.13~ 0.15)	0.86
Time * SES-CD		
Time* mild/moderate	-0.03 (-0.06~ 0.00)	0.032
Time* severe	0.00 (-0.04~ 0.05)	0.89

**Characteristics**	**Beta (95% CI)**	**p-value**
time	0.05 (0.01~0.08)	0.02
Sex		
F	-	
M	0.33(0.13~0.54)	0.002
CT activity		
Normal	-	
Mild	0.09(-0.24~0.42)	0.59
Moderate	0.08(-0.16~0.31)	0.51
Severe	0.15 (-0.07~0.37)	0.19
Time * CT activity		
2-5y f/u	0.0 (-0.06~ 0.06)	0.88
5-8y f/u	-0.04 (-0.08~0.01)	0.15
>8y f/u	-0.04 (-0.08~0.00)	0.062

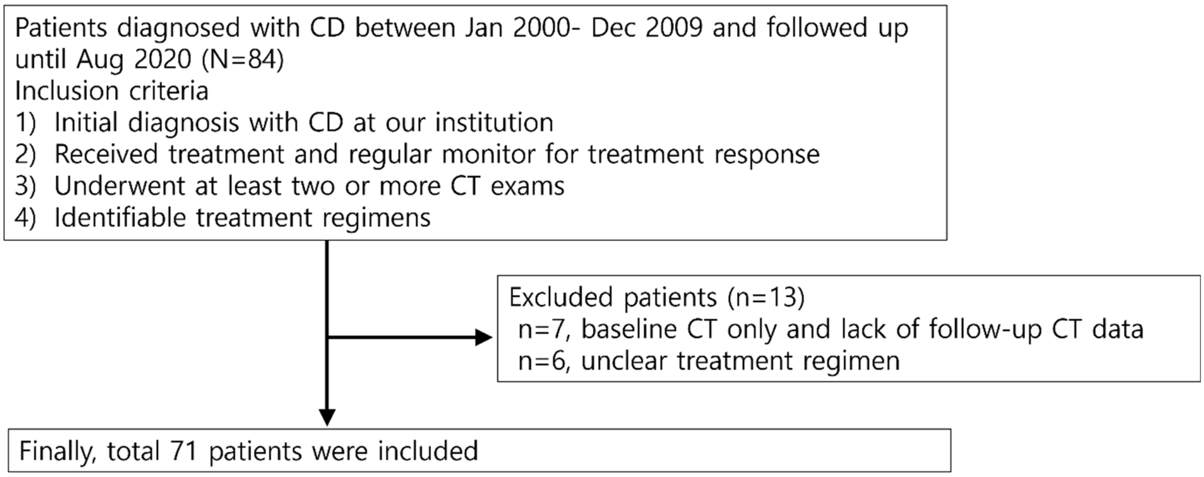

Supplementary Figure S1. Flow chart of patient selection.

1) CT activity grades

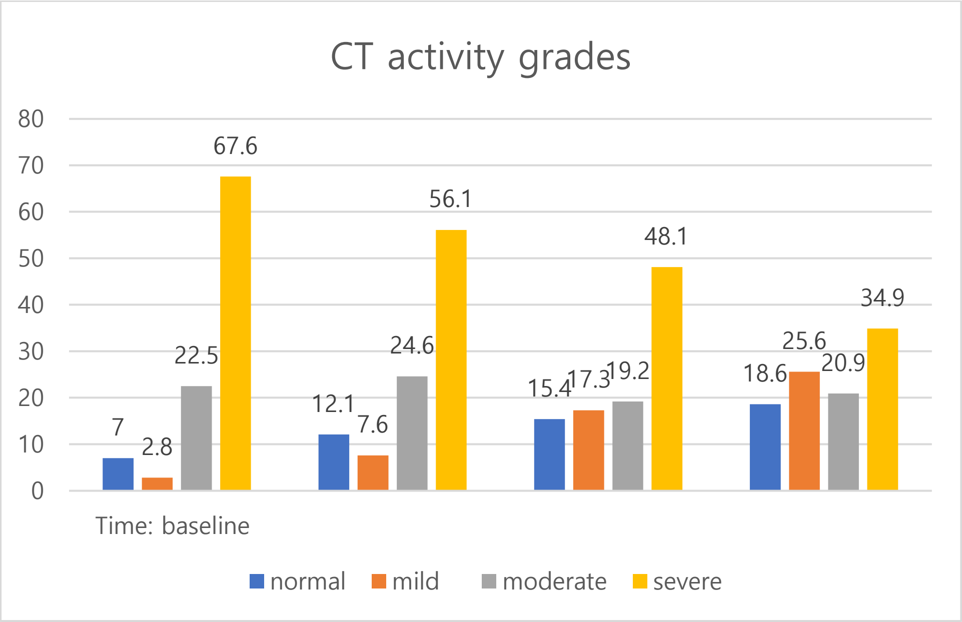

2) SES-CD scores

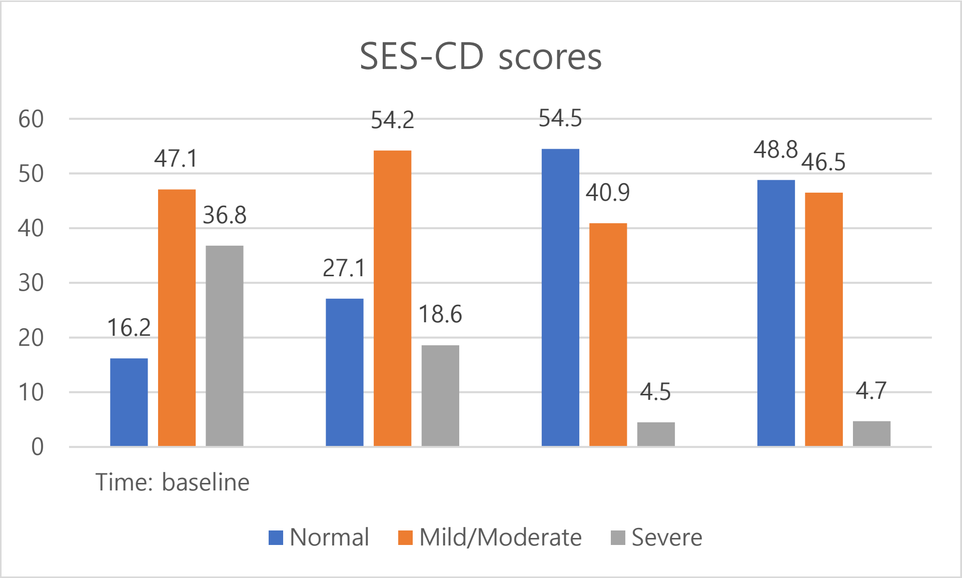

Supplementary Figure S2. Changes in disease activity.
